# Autoimmune CD8+ T cells in type 1 diabetes: from single-cell RNA sequencing to T-cell receptor redirection

**DOI:** 10.3389/fendo.2024.1377322

**Published:** 2024-05-10

**Authors:** Kangping Yang, Yihan Zhang, Jiatong Ding, Zelin Li, Hejin Zhang, Fang Zou

**Affiliations:** ^1^ Department of Endocrinology and Metabolism, The Second Affiliated Hospital of Nanchang University, Nanchang, China; ^2^ The Second Clinical Medicine School, Nanchang University, Nanchang, China; ^3^ The First Clinical Medicine School, Nanchang University, Nanchang, China

**Keywords:** type 1 diabetes, CD8+ T cells, single-cell RNA sequencing (scRNA-seq), CRISPR/Cas9, chimeric antigen receptor T-cell (CAR-T)

## Abstract

Type 1 diabetes (T1D) is an organ-specific autoimmune disease caused by pancreatic β cell destruction and mediated primarily by autoreactive CD8+ T cells. It has been shown that only a small number of stem cell-like β cell-specific CD8+ T cells are needed to convert normal mice into T1D mice; thus, it is likely that T1D can be cured or significantly improved by modulating or altering self-reactive CD8+ T cells. However, stem cell-type, effector and exhausted CD8+ T cells play intricate and important roles in T1D. The highly diverse T-cell receptors (TCRs) also make precise and stable targeted therapy more difficult. Therefore, this review will investigate the mechanisms of autoimmune CD8+ T cells and TCRs in T1D, as well as the related single-cell RNA sequencing (ScRNA-Seq), CRISPR/Cas9, chimeric antigen receptor T-cell (CAR-T) and T-cell receptor-gene engineered T cells (TCR-T), for a detailed and clear overview. This review highlights that targeting CD8+ T cells and their TCRs may be a potential strategy for predicting or treating T1D.

## 1 Introduction

Type 1 diabetes (T1D) is an autoimmune disease in which T lymphocyte-mediated pancreatic β cell failure occurs and patients are dependent on exogenous insulin therapy for life (1). T1D affects millions of people worldwide and is continuing to increase by 3-4% per year (2). Its multiple acute complications, long-term complications, other autoimmune diseases and psychosocial problems have an enormous impact on the survival of patients (3). Currently, T1D is only treated with insulin therapy, pancreas transplantation or islet transplantation, which are the more limited treatments. Islet transplantation and pancreas transplantation are associated with high surgical risks, inadequate organ sources, high financial stress, limited survival of islet cells after transplantation and insufficient ability to correct blood glucose (4). Insulin therapy has none of these risks but requires multiple daily injections and difficult glycemic control, eventually leading to complications and premature death (5, 6).

β cell-specific CD8+ T cells are considered a new direction for long-acting immunotherapy against precise targets, which is likely to be a key breakthrough point for T1D treatment (6). There are many current immunotherapeutic ideas and targets for T1D, such as anti-CD3 antibodies (7), immune checkpoint inhibitors (8), anti-thymocyte globulin antibodies (9) and other drugs, but they have been plagued by off-target effects with long-term failure. With the advancement of research, β cell-specific CD8+ T cells in pancreatic draining lymph nodes (pLN) have been valued (10, 11). A population of β cell-specific CD8+ T cells with stem cell characteristics exists in the pLN and is capable of long-term survival and differentiation of destemmed β cell-specific CD8+ T cells that are continuously delivered into the pancreas to produce sustained autoimmune killing. Transferring hundreds of thousands of intrapancreatic T cells from T1D mice to new mice fails to cause T1D, whereas only 20 stem cell-like CD8+ T cells at the pLN are needed to cause T1D in healthy mice (12). Therefore, the use of β cell-specific CD8+ T cells may allow for precise and sustained treatment of T1D.

T-cell recognition of antigens depends mainly on the T-cell receptor (TCR) on its surface, and different clones of T cells have different TCR sequences, which constitute a highly diverse TCR pool in the body in response to external antigens, and the TCR determines the specificity of CD8+ T-cell recognition for killing (13, 14). Therefore, many studies have attempted to use single-cell RNA sequencing (ScRNA-Seq) technology for high-throughput screening of pathogenic TCR target platforms, CRISPR/Cas9 gene editing technology has been applied to T-cell transformation, and chimeric antigen receptor T-cell (CAR-T), T-cell receptor-gene engineered T cells (TCR-T), and other technologies have been focused on in the treatment of T1D.

At present, there are many still unclear aspects of β cell-specific CD8+ T cells in T1D, such as the heterogeneity of CD8+ T cells (15) and the process of T-cell differentiation (16) still remain to be explored. These unclear issues affect the depth of related research; therefore, this paper will provide a detailed and clear review of the mechanism of autoimmune CD8+ T cells and TCR in T1D, as well as the important applications of scRNA-seq, CRISPR/Cas9, CAR-T and TCR-T.

## 2 Adaptive Immunity in T1D

### 2.1 The pathogenesis of T1D

**Figure 1:**
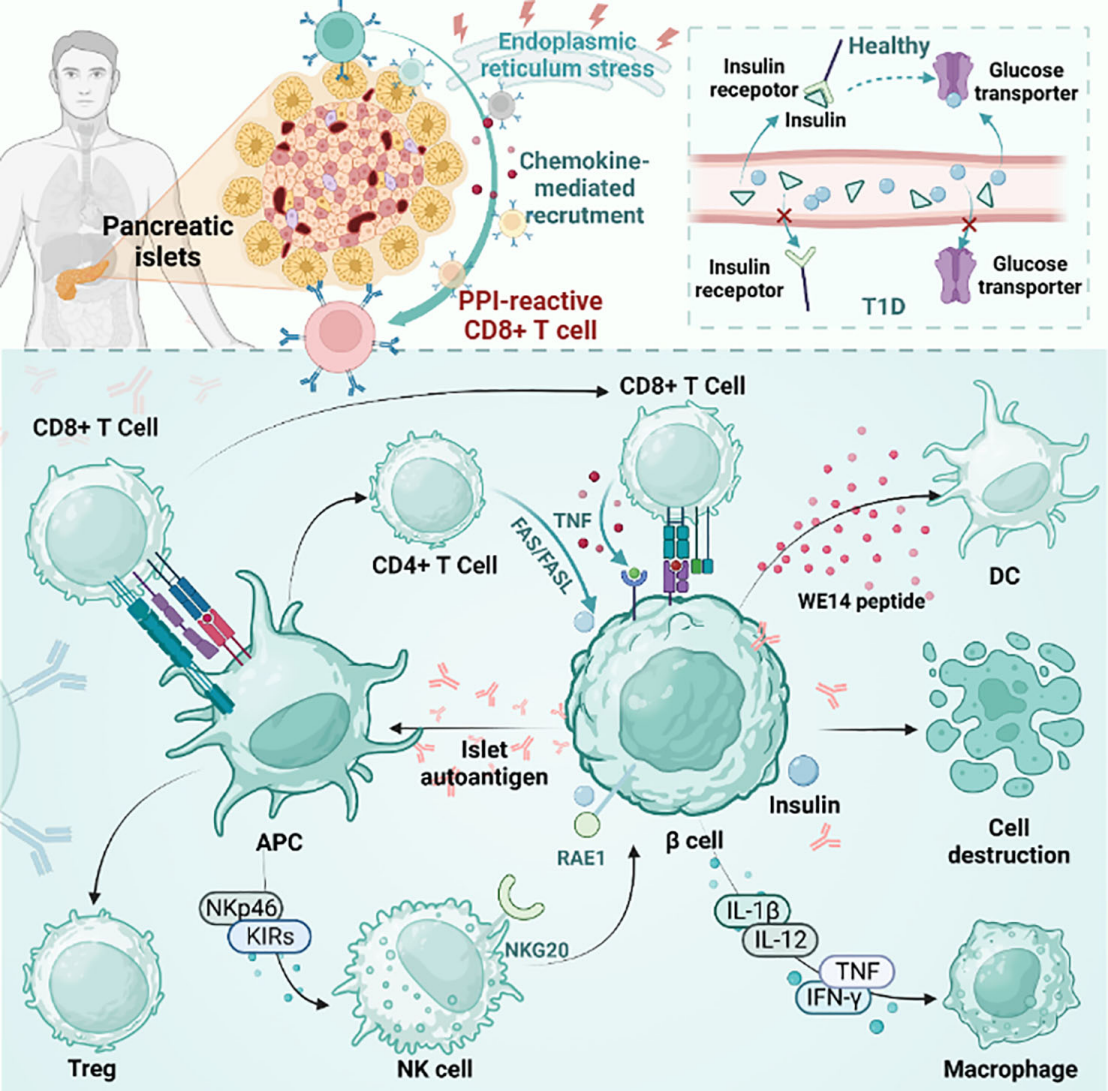
The pathogenesis of T1D. The figure plots the differences between T1D and healthy individuals and the mainstream accepted pathogenesis of T1D. pLN, pancreatic draining lymph node; APC, antigen presenting cells; PD-1, programmed death-1; CTLA-4, cytotoxic T lymphocyte-associated antigen-4; TCF1, transcription factor 1; Treg, regulatory T cell; NK cell, natural killer cell; DC, dendritic cell; PPI, preproinsulin.

The pathogenesis of T1D is complex, involving several different immune cell subsets, pathways, and complex islet autoimmune responses (6). CD4+ and CD8+ T cells are involved in the development of T1D, and autoimmune T cells directly recognize β cell autoantigens on antigen-presenting cells and rapidly transform into effector T cells, while CD8+ T cells are the main killers of islet β cells (**Figure 1**) (17).

However, the exact mechanism behind the failure to turn on immune tolerance to β cell autoantigens in the common T1D pathogenic mechanism is a mystery (18). The pathogenesis of human islet disease remains unclear, but pancreatic remodeling has been shown to be associated with it in NOD model mice, which directly promotes β cell apoptosis and antigen release. The released antigen is phagocytosed by macrophages and DCs. These APCs are then transported to draining popliteal lymph nodes (pLNs) to promote β cell-specific T-cell generation and promote effector T-cell differentiation. After that, the effector T cells mature and travel to the islets to promote inflammation, which leads to islet inflammation (19–22). It is worth mentioning that many studies have shown that thymus selection disorder is a specific indication of islets in NOD mice (22, 23).

In normal people, insulin production is responsible for pancreatic beta cells, and insulin plays an important physiological role in the body. Insulin activates downstream signaling pathways by binding to insulin receptors on the cell surface, thereby regulating glucose metabolism and utilization. Compared with nondiabetic individuals, pancreatic beta cells in genetically susceptible individuals with T1D are selectively destroyed and cannot effectively produce enough insulin. This means that the insulin receptors are unable to bind to enough insulin, causing downstream signaling pathways to not open properly. As a result, glucose cannot be effectively absorbed and used by cells, leading to hyperglycemia (24). Healthy human pancreases contain self-reactive CD8+ T cells, which increase in number during the development of diabetes. Among autoreactive CD8+ T cells, preproinsulin (PPI)-reactive CD8+ T cells were more common in T1D patients than in healthy donors (25). PPI-reactive CD8+ T cells had a similar frequency in the exocrine pancreas regardless of the disease state. Optimal staining with PPI tetramer isolates functional T cells. However, during disease progression, PPI-reactive CD8+ T cells are attracted to islets. Under physiological conditions or stress, cytokine secretion may contribute to immune cell recruitment and β cell killing (26). β cells are destroyed by various factors and release autoantigens that are presented by antigen-presenting cells. CD4+ T, CD8+ T and NK cells are then activated. In addition, NK cells are involved in the direct killing of β cells through the interaction of NK cell markers such as NKp46 and KIRs. In addition, CD8+ T cells promote the development of T1D by secreting proteins such as Fas and cytokines such as TNF-α and IFN-γ (27). Cytokines secreted by natural killer (NK) cells and immune cells directly damage beta cells while also inducing self-defense mechanisms. Cytokines include interleukin-1β (IL-1β), IL-12, interferon-γ (IFN-γ), and tumor necrosis factor (TNF). Immune cells include macrophages, DCs, CD4+ T cells, and CD8+ T cells. In nonobese diabetic (NOD) mice, NKG2D expressed by NK cells binds to RAE1 expressed by β cells and is associated with β cell damage. In older transgenic mice that are not controlled, the expression of RAE1 is sufficient to induce the recruitment of adaptively transferred cytotoxic T lymphocytes (CTLs) to islets, resulting in the recruitment of a large number of endogenous lymphocytes. Eventually, it leads to pancreatitis. This is dependent on CTL expression of NKG2D, independent of antigen recognition (28). Islet β cell antigens are also presented by MHCII molecules expressed by antigen presenting cells. T cells expressing FasL can mediate apoptosis by interacting with FAS expressed on β cells (29).

### 2.2 Current immunotherapy strategies

The only current treatment for T1D is insulin replacement. Currently, combined immunotherapy has become a new trend in the treatment of T1D. Immunotherapy, such as the use of monoclonal antibodies, is a strategy to target specific populations of immune cells that induce autoimmunity to drive pathology. For example, monoclonal antibodies (mAbs) have been shown to have no side effects or positive effects in regulating T1D (30). Previous studies have found that anti-CD3 monoclonal antibodies reduce the loss of insulin production and the need for exogenous insulin to maintain glycemic control during the first 2 years of T1D (31). There are two main approaches to the main immune treatment of T1D. The first is to block the pathogenic response by antagonizing B cell, cytokine and T-cell activation. The other promotes immune regulation by restoring or promoting regulatory T-cell function, promoting depletion of T-cell generation and β cell regeneration (6). Clinical data suggest that this combination therapy is more suitable for the treatment of this chronic disease than other therapies (32). Today, secondary prevention, which uses immunotherapy to successfully delay preclinical disease progression, and primary prevention, which suppresses autoimmune initiation, have entered large-scale clinical trials (**Table 1**). By shifting the focus of T1D treatment from late diagnosis and insulin replacement to early diagnosis and β cell preservation, children with T1D could dispense daily insulin injections in the future (6, 33). Strategies based on immunotherapy targeting the blocking of T-cell responses to β cell autoantigens have recently been incorporated into a group of existing T1D therapies, which are beneficial for blocking the onset and development of T1D. In recent years, new breakthroughs have been made in preserving the activity of islet beta cells. Chen et al. achieved the goal of generating pancreatic β-like cells from gallbladder stem cells (GSCS) without genetic modification by screening the combination of small molecules that produce insulin-secreting cells from gallbladder stem cells (GSCS) (34, 35). The small molecule combinations they found were Noggin, FR180204 and cyclopamine, and the addition of this combination could effectively induce the differentiation of gallbladder stem cells into insulin-secreting cells. In addition, targeting MHC class II molecular proteins is a possible way to alleviate T1D, for example, by activating cathepsin G (CatG) to degrade MHC class I molecules to attenuate CD4+ T-cell activation in NOD mice and improve islet function (36–38).

**Table 1:** Prevention of T1D - non-antigen-specific immune interventions.

Medicine	Patient age group (years)	Target point	Phase	References
ATG	12 to 45 (Children, adults)	T cells	Stage 2	NCT02215200
Abatacept (CTLA4-Ig)	6 to 45 (Children, adults)	T-cell activation: CD80, CD86	Stage 2	NCT00505375
Alefacept	12 to 35 (Children, adults)	T cells (CD2)	Stage 2	NCT00965458
Anti–IL-21 (NNC0114-0006) (+ liraglutide)	18 to 45 (Adults)	IL-21 (T cells, B cells, natural killer cells)	Stage 2	NCT02443155
Rituximab	8 to 40 (Children, adults)	B cells (CD20)	Stage 2	NCT00279305
Golimumab	6 to 21 (Children, adults)	TNF	Stage 1	NCT03298542
Teplizumab	0 to 7 (Children)	T cells (CD3)	Stage 4	NCT05757713

Prevention of T1D by restoring or inducing immune tolerance to β cells is the main measure against T1D in the future (18). The use of antigen-specific immunotherapy can restore a self-tolerant immune system in which T-effector cells are suppressed and/or T-regulatory cells are induced (39). It is well known that some defects in innate and adaptive responses of the immune system lead to an imbalance in the regulation of autoimmune responses. Tregs play an important role in regulating disease progression because they can inhibit any inappropriate autoimmune response. Several interventions have been developed based on this finding (40, 41). Amatya et al. demonstrated the possibility of anti-T1D therapy by constructing a cell-permeable PDX1-FOXP3-TAT fusion protein (FP) to stabilize Tregs for the purpose of anti-autoimmune and insulin production (42). The protein was tested both *in vitro* and *in vivo* (nonobese diabetic mouse T1D model). *In vitro*, FP transforms naive CD4 T cells into a functional “Treg-like” subpopulation that inhibits cytokine secretion and downregulates antigen-specific responses. In liver stem cell-like cells, increased endocrine transdifferentiation and increased expression of insulin 2 and other β lineage-specific genes were observed. *In vivo*, the following results were observed when the protein was administered to T1D model mice: significant increases in insulin and C-peptide levels, the formation of insulin-containing cell clusters in the liver, and systemic anti-inflammatory transformations. In addition, targeting islet-specific Tregs is more effective than targeting polyclonal Tregs in the prevention of T1D. In fact, the frequency of innate antigen-specific Tregs is extremely low, and expansion *in vitro* predisposes Tregs to impaired stability, leading to an effector phenotype. Yang et al. used homology-directed repair to increase the expression of FOXP3 in Tregs and performed lentiviral vector-based human T-cell receptor gene transfer to improve the specificity of Tregs for β-islet cells in T1D. It promotes the generation of islet-specific Tregs and inhibits effector T-cell proliferation and cytokine production, thereby blocking diabetes triggered by islet-specific T_eff_ or diabetogenic polyclonal T_eff_ (43). Adjuvant immunotherapy upregulates regrowth (Reg) genes in islets and induces Th17 cells that produce interleukin 22 (IL-22). Previous studies have revealed that IL-22 upregulates _reg_ gene expression in pancreatic islets and may induce β cell regeneration and prevent apoptosis (44–46). Furthermore, animal experiments have shown that IL22 expression helps to reduce the severity of streptozotocin diabetes in mice fed a grain-fed diet (47). IL-2 can be used to treat T1D because it promotes immunity or tolerance depending on its availability. Blockade of the T_eff_ cytokine IL-2 can be used to prevent and treat T1D in NOD mice (48). Low doses of IL-2 (LD IL-2) are known to drive tolerance by preferentially acting on Tregs, thereby providing immune modulation with few side effects. Tregs are an IL-2-reactive cell type known to control autoimmunity. Treatment with LD IL-2 in patients with immune-mediated diseases increases T _reg_ numbers and controls autoimmunity. However, it has also been found that patients have defective or defective IL-2 production or signaling and that IL-2 treatment causes mild activation of NK cells and eosinophils (49). According to research, regulatory T cells (Tregs) prevent the targeting of autoantigens by T_eff_ cells. Therefore, it is possible to enhance the frequency and function of antigen-specific Tregs by adding low doses of IL-2 to the antigen therapy regimen (50). Cytokines such as IL-6 at physiological levels can maintain glucose homeostasis in islet cells, and in addition, other cytokines such as IFN-γ and CXCL10 may play a pathogenic role by promoting immune cell recruitment and β-cell killing (51). Islet cells express a wide range of cytokine receptors, such as IL-4R (52, 53), IL-13R and IL-6R. Therefore, cytokines can both induce and regulate T1D and have the potential to regenerate and preserve insulin-producing β cells in islets (26, 46).

In addition, important breakthroughs have also been made in targeted therapy for T1D. Recent studies have shown that decreased GLUT4 expression/translocation is associated with impaired glycemic control in diabetes, specifically affecting insulin-induced glucose uptake in muscle and adipose tissues (54). It has also been reported that gene therapy was used to reestablish central tolerance in NOD mice by reestablishing hematopoietic stem cells (HSCs) with retroviral transduction to express the MHC class II β chain in a protective form (55). In addition to the methods mentioned above, organ transplantation is the last option. Islet and pancreas transplantation in humans could alleviate the dilemma faced by patients with diabetes, but its use has thus far been limited by organ scarcity and lifelong administration of immunosuppressive drugs. However, recent studies have proposed the latest method to efficiently generate pancreatic progenitor cells (PPs) and β-like cells from human pluripotent stem cells (hPSCs) *in vitro*, which is expected to improve the cure rate of diabetes (56). On the whole, new T-cell techniques hold promise for defining the process of autoimmune T-cell differentiation and characterizing autoimmune responses in comparison to physiological immune responses (18).

### 2.3 Potential breakthrough point for targeted therapies——CD8+ T cells

The pathogenesis of T1D involves immune regulation and immune response, in which cellular immunity plays a key role. Cellular immunity is a major killer of islet β cell destruction. Pancreatic β cell damage is influenced by genetics, environment, and immunity, and genetic and environmental factors increase T1D risk by partially altering central and peripheral tolerance-inducing events. In addition, 50% - 60% of the genetic risk of T1D comes from HLA alleles that encode molecules involved in the presentation of antigenic peptides to T cells. Regrettably, these effects on antigen presentation greatly affect thymus selection processes and peripheral activation of immune responses (57, 58). For example, mutations in the STAT3 gene can cause the body to stop tolerating CD8+ T cells, causing disease (59). It was thus found that diabetes-induced CD8+ T-cell responses are restricted in the presence of normal STAT3 activity and drive diabetic pathogenesis. At the same time, overexpression of class I HLA in islet cells in T1D is closely related to increased expression of STAT1, resulting in selective susceptibility to the occurrence of autoimmune diseases in the population (60). Apart from HLA-I, HLA-II also contributes to the development of T1D. HLA-II is also expressed in pancreatic β cells of patients with T1D, and HLA-II-expressing β cells may be the direct target of autoimmune CD4+ T cells (61, 62). Therefore, HLA-II also plays a role in the pathogenesis of T1D (62). Individual HLA differences also affect peptide binding and signal transduction after TCR conjugation. This drives the development and expansion of β cell-specific effector T cells for islet inflammation (22, 27).

Despite differences between people, CD8+ T cells have been shown to be the dominant immune cell type in islet lesions, followed by CD68+ macrophages, CD4+ T cells, and CD20+ B cells (63). It has been shown that the critical subgroup of dendritic cells responsible for the cross-presentation of islet antigens with CD8+ T cells and the direct presentation of β cell antigens to CD4+ T cells, termed merocytic dendritic cells (mcDC), are more numerous in nonobese diabetic (NOD), are typical of critical antigen presenting cells and are responsible for disrupting peripheral tolerance to β cell antigens *in vivo* (64).

In general, the islet β cell autoimmune response has progressed for several years prior to the clinical diagnosis of T1D (65). Given that autoimmune T cells are present in both patients with autoimmune diseases and healthy individuals, whether an individual develops autoimmunity depends on the balance between these potentially pathogenic self-reactive immune cell types and the regulatory mechanisms that control them (40). The best therapy for T1D prevention and treatment strategies of islet replacement or regeneration is to tolerate or suppress the autoimmune T-cell response while keeping the immune system functioning well in response to foreign antigen invasion and avoiding the use of global immunosuppression. In addition, it is now widely accepted that autoantigen-driven T-cell clonal amplification is the hallmark of T1D pathophysiology. Examination of TCR clones amplified from T1D donor pancreases produced mixed but generally positive results. This will also become a breakthrough point for future targeted therapy (66). The TCR clones expanded from the T1D donor pancreas produced mixed but overall positive results, implying that in the pancreas of T1D patients, there are multiple T-cell clones that react with autoantigens of the same epitope. These TCR clonal sequences can be used therapeutically because they can be used to develop immunotherapies targeting specific antigens to reduce or eliminate autoimmune responses. Specifically, these TCR clonal sequences can be used to develop antigen-specific T-cell immunotherapies, such as CAR-T-cell therapies.

## 3 Autoimmune CD8+ T cells in T1D

Antigen-specific CD8+ T cells include effector memory T cells, central memory T cells, peripheral memory T cells, tissue-resident memory T cells, and stem cell memory T cells. T_eff_, T_cm_, and T_scm_ cells are peripheral memory T cells, while tissue-resident memory T cells are mainly T_rm_ cells. In T1D, the proportion of peripheral memory T cells and tissue-resident memory T cells changes with time and Ag-encounter. Specifically, Trm cells are mainly found in tissues such as the pancreas and intestine, while T_cm_ and T_scm_ cells are mainly found in lymph nodes and peripheral blood. In the early stages of T1D, the proportion of peripheral memory T cells is higher, and the proportion of tissue-resident memory T cells is lower. As the disease progresses, the proportion of tissue-resident memory T cells gradually increases, probably because they are better able to localize and function in the pancreas. Therefore, the changes in the proportions of T_eff_, T_cm_ and T_scm_ cells, as well as T_rm_ cells, in T1D are dynamic and may be influenced by a variety of factors (67). The environment of early naive T cells can change the activation state of CD8+ T cells, leading to memory T cells, exhausted T cells, senescent T cells, and so on. In particular, CD8+ T cells regulate Fas-FasL-mediated killing of islet β cells, mainly through the homologous interaction between perforin and granzyme B. (48). The FasL-Fas interaction plays an important role in regulating CD8 expression. The expression of Fas and FasL is regulated by cell activation signals, and the expression levels of Fas and FasL in CD8+ T cells gradually increase after cell activation. Specifically, Fas transcription began at 24 h after cell activation and reached an approximately 8-fold increase at 72 h. In contrast, FasL transcription appears earlier after cell activation and reaches higher levels in cells stimulated by anti-CD3 and anti-CD28. At the protein level, the expression of Fas and FasL also reflected its regulation at the mRNA level. Fas was mainly induced by BM-DCs, and its level gradually increased during the observation period. In contrast, FasL was similarly induced by the combined stimulation of BM-DCs and anti-CD3 and anti-CD28 antibodies. These results suggest that Fas and FasL expression in CD8+ T cells is regulated, at least in part, at the transcriptional level and influenced by signals present during cell activation (68).

**Figure 2:**
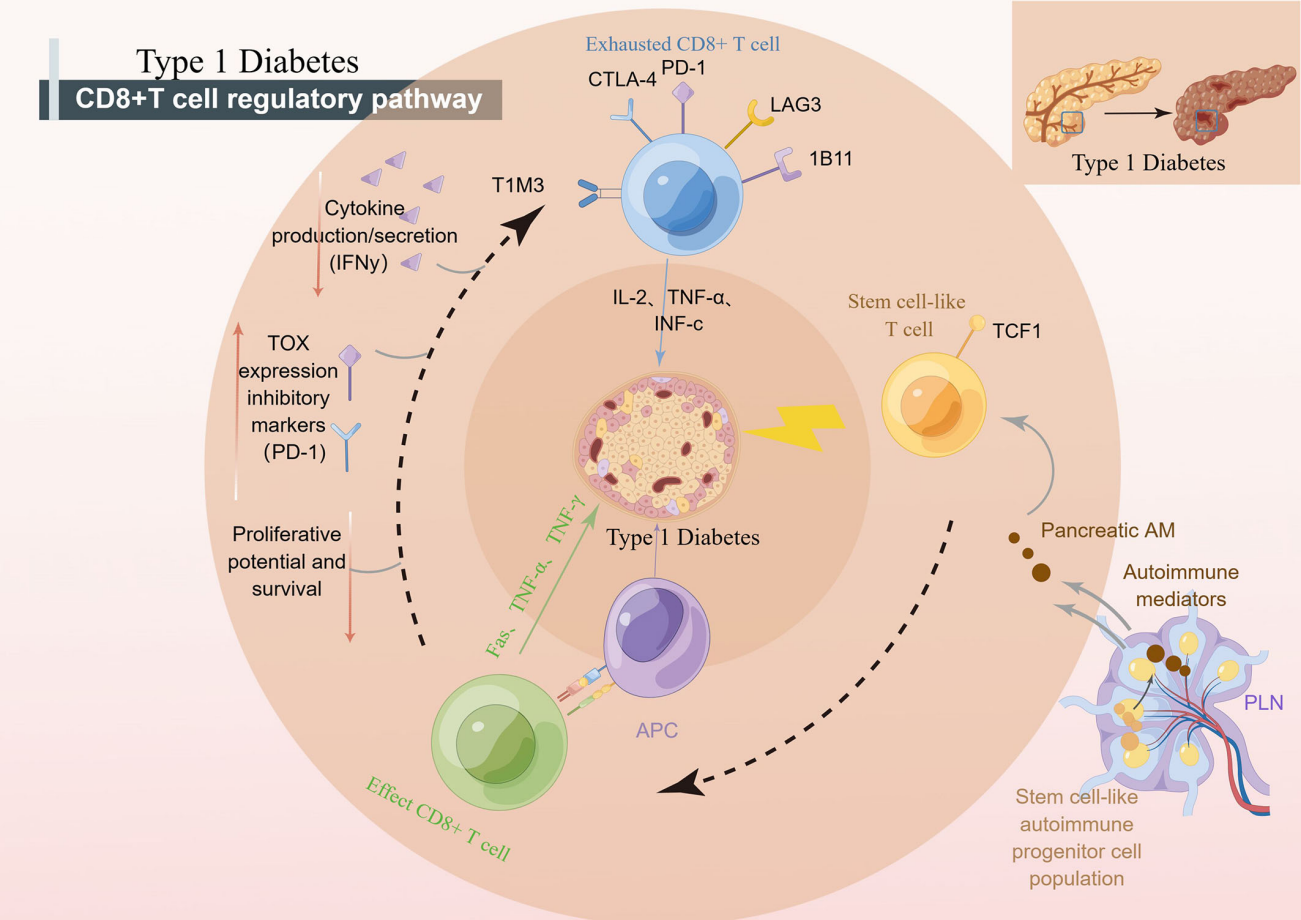
CD8+ T cells regulate the development of T1D. pLN, pancreatic draining lymph node; APC, antigen presenting cells; PD-1, programmed death-1; CTLA-4, cytotoxic T lymphocyte-associated antigen-4; TCF1, transcription factor 1; NOD, nonobese diabetic.

Antigen-specific T lymphocytes kill insulin-producing β cells in T1D (T1D) by disrupting central and peripheral tolerance (64). Among these killers, CD8+ T cells are crucial for clearing many bacterial or viral illnesses because they kill infected cells (69). CD8+ T cells have a variety of functional and developmental states, including effector cells, regulatory cells, and hypofunctional states. In T1D, circulating islet antigen-specific CD8+ T cells exhibit a wide range of phenotypic heterogeneity, including an early memory phenotype, a stem cell memory phenotype, a transitional memory phenotype, a final effector memory phenotype, and a hypofunctional state (70). One of them is CD8 memory T cells. An initial expanded vaccination regimen can result in multiple contacts with Ag over the host’s lifetime due to recurrent exposure to the same pathogen, which increases the amount of CD8 memory T cells (67, 71). Effector CD8+ cells are another state. The recognition of cytotoxic T cells by islet autoantigens provided by class I HLA molecules may be a crucial effector mechanism that results in the killing of β cells in effector CD8+ cells, according to pertinent research (41). CD8+ T cells with declining function have come into the public eye in recent years. In studies, T-cell populations characterized by inhibitory receptors and inhibitory receptor-mediated depletion were closely associated with improved T1D markers (72). Inhibitory receptors are a class of proteins that play an important role in regulating the immune response. Inhibitory receptor-mediated depletion refers to the gradual loss of effector function of T cells and the continuous upregulation of a variety of inhibitory receptors in the context of chronic infection, cancer immunotherapy, and autoimmunity. This depletion state makes T cells unable to kill target cells effectively, which leads to disease progression. In the treatment of T1D, the role of inhibitory receptors is to inhibit the autoimmune response by inducing the exhaustion state of T cells, reduce the attack on islet β cells, and protect islet function. An example is the PD-1 (programmed death 1) inhibitory receptor. PD-1 is a receptor expressed on the surface of activated T cells. When it binds to its ligand PD-L1 (programmed death ligand 1), it can inhibit the activation and function of T cells. In T1D, the activation of PD-1 inhibitory receptors can reduce the autoimmune response, reduce the attack on islet β cells, and thereby protect islet function. Therefore, where are the reduced T cells going to come from? Kong et al. showed that autoimmune tissue could maintain an undifferentiated central memory-like autoimmune T-cell pool, which has the potential for pathogenic effects and may be an important source of effector T cells during long-term chronic autoimmunity (73). It is worth mentioning that stem-like CD8+ T cells, effector CD8+ T cells and depleted CD8+ T cells can coexist in the pancreas of patients with TID (**Figure 2**). Stem-like CD8+ T cells, effector CD8+ T cells, and exhausted CD8+ T cells can coexist in the pancreas of TID patients. These different types of CD8+ T cells play different roles in the development and progression of TID. Stem cells, such as CD8+ T cells, can self-renew and differentiate into T cells but can also remain in a more primitive state. Effector CD8+ T cells can kill beta cells in the pancreas, resulting in reduced insulin secretion. Exhausted CD8+ T cells lose their ability to kill beta cells, possibly due to prolonged antigenic stimulation. The coexistence of these CD8+ T cells may be an important feature of the complex pathological process of TID (74).

### 3.1 Stem-like CD8+ T cells

Recent studies suggest that CD8+ stem-like T cells may play an important role in the pathogenesis of T1D. It has been found that effector T cells during long-term chronic autoimmunity may originate from an undifferentiated central memory-like autoimmune T-cell pool in autoimmune tissues, which contributes to the disease (73). T cells have the characteristics of stem cells and are an important cell type in the immune system. According to their stem cell properties, T-cell stemness often segregates T cells into short-lived and terminally differentiated effector cells and long-lived progenitors that can give rise to terminally differentiated T effectors. In addition, the transcription factors TCF1, Bach2, c-MYB, and Foxo1 were found to be critical for the induction of T-cell stem cell properties (75). Among them, the autoimmune T cells that express TCF1 in pLN are stem-like progenitor cells that can drive T1D by generating short-lived TCF1-T factor cells that destroy pancreatic β cells. Therefore, targeting stem cell-like progenitor cells may be a new breakthrough for the treatment of T1D (75, 76).

In T1D, beta cell-specific CD8+ T cells destroy insulin-producing beta cells. A group of β-specific CD8+ T cells in pLN with stem-like characteristics continue to differentiate and import β-specific CD8+ T cells into islets, leading to long-term β cell damage (76). Recent studies have shown that weak MHC-infiltrating CD8+ T cells from islets may, through presentation, lead to weak TCRs, thereby maintaining some stem-like phenotypes of islet-reactive CD8+ T cells (77). “Weak MHCI-infiltrating CD8+ T cells” refers to the ability of specific types of CD8+ T cells (cytotoxic T cells) to infiltrate cells that express lower levels of major histocompatibility complex class I (MHCI) molecules in an immune response. However, some CD8+ T cells also have the ability to infiltrate cells that express lower levels of MHC I molecules. These cells are called “weak MHCI-infiltrated CD8+ T cells”. They can recognize and attack cells through other pathways that do not rely on antigen presentation by MHC I molecules. This allows them to mount an immune response against certain cells that express lower levels of MHC I molecules, such as certain tumor cells or infected cells. Abdelsamed et al. assessed the pluripotency index of DNA methylation in T cells and found that β cell-specific CD8+ T cells had a stem-like epigenetic pluripotency score. Abdelsamed et al. demonstrated that autoimmune CD8+ T cells isolated from lymphoid tissues retained a developmentally plastic phenotype and epigenetic signatures compared with the same cells isolated from the pancreas (13). In other words, a subset of cells in CD8+ T cells with stem cell characteristics is strongly associated with the progression of T1D. When efficacy is enhanced by epigenetic mechanisms, patient tolerance can be improved by inducing CD8+ T cells with stem cell characteristics to enter a tolerance state (13). T_scm_ cells have stem cell properties and are the earliest developmental stage of memory T cells. Stem cell T cells specific for β cell autoantigen protein, insulin, and islet cell-specific glucose-6-phosphatase-catalyzed subunit associated protein (IGRP) were found in patients with autoimmune CD8+ T cell. Autoimmune T_scm_ in T1D patients can selectively target autoantigens by inhibiting glucose metabolism (78). Researchers are now further investigating the specific mechanisms and functions of CD8+ stem-like T cells in T1D. Understanding the role of these cells may help shed light on the pathogenesis of T1D and provide new targets for the development of new therapeutic strategies. However, there are still many unknown aspects of the role of CD8+ stem-like T cells in T1D, and further studies are needed to deepen our understanding.

### 3.2 Effector CD8+ T cells

T_eff_ plays a major role in the pathogenesis of T1D, and T-cell recognition, activation, expansion, and function influence active immune processes (77). T_eff_ can be divided into several subsets based on their different characteristics (77, 79). Effector T cells can be divided into several different subsets as follows: CD4+ T cells are a class of T cells with CD4 surface markers that regulate and coordinate immune responses primarily by producing a variety of cytokines. CD4+ T cells can be further divided into Th1, Th2, Th17 and T _reg_ subgroups. CD8+ T cells: CD8+ T cells are a class of T cells with CD8 surface markers that are primarily responsible for directly killing infected cells and tumor cells. CD8+ T cells exert their effects by releasing cytotoxins and producing cytokines (77, 79). Natural killer T cells (NKT cells): NKT cells are a class of T cells with natural killer activity that can rapidly recognize and kill infected cells and produce a variety of cytokines to regulate the immune response. Gamma delta T cells: Gamma delta T cells are a class of T cells with a gamma delta T-cell receptor whose structure is different from that of the traditional alpha beta T-cell receptor. γδT cells can directly recognize and kill infected cells and play an important immunomodulatory role. These different effector T-cell subsets have different functions and roles in the immune response and cooperate with each other to exert important immune effects (80). In nonobese diabetic (NOD) mouse models, CD8+ T effector cells play a key role in islet β cell destruction and contribute to the maintenance of islet inflammation (24, 40). Patients with T1D exhibit impaired peripheral tolerance, including T _reg_ hypofunction and resistance of effector T_eff_ cells to regulation by Tregs; that is, effector T cells in patients with diabetes are not sensitive to regulation by CD4+ FOXP3+ regulatory T cells (80). T_eff_ targets a group of epitopes derived from islet proteins, including proinsulin, GAD, IA-2 and IAPP. Studies have shown that with the appearance of neoepitopes one after another, which are different from the conventionally modified epitopes, the generation of neoepitopes will lead to the activation of pathogenic immune cells, thereby starting a feedforward circuit that can amplify the antigen repertoire against pancreatic β cell proteins and lead to T1D (81, 82). Interestingly, T_eff_ resistance is not associated with a specific subset or marker but rather with the activation status of T_eff_ and exposure to proinflammatory cytokines, particularly IL-6. During the development of human T1D, T_eff_ resistance appears to be STAT3 dependent but not directly associated with the capacity of T cells to produce or respond to IL-6. (32, 35, 83, 84). The resistance of effector T cells to the inhibition of regulatory T cells is one of the characteristics of the development of diabetes, and this resistance is related to the STAT3 signaling pathway but not to the ability of effector T cells to produce or respond to IL-6. Studies have shown that T1D is more likely to develop in children than adults due to a more amplified T-cell response to β cell autoantigens (85).

### 3.3 Exhausted CD8+ T cells

In recent years, exhausted CD8+ T cells have been a research hotspot in the fields of autoimmunity, such as T1D, tumor immunotherapy and chronic inflammation (72). CD8+ T-cell exhaustion refers to the transition from precursor cells to terminally exhausted cells, which is a process of continuous change (86). On the basis of studies in cancer and chronic viral infections, a three-signal model for the development of T-cell failure has been proposed, namely, continuous antigenic stimulation, negative costimulatory signaling, and chronic inflammation (87). In this process, T cells exhibit a variety of cellular and molecular characteristics, such as loss of T-cell effector function, cytokine response, metabolism, gene expression, and epigenetic changes (35, 87). Thus, the transcriptional signature of CD8+ T cells is one of the determinants of good prognosis in a variety of autoimmune diseases, especially systemic diseases (72, 87). The transcriptional characteristics of CD8+ T cells include overexpression of various inhibitory receptors, such as PDCD1, KLRG1, TIGIT, HAVCR2 (TIM3), LAG3, CTLA4, CD160, and CD244. The coexpression of these inhibitory receptors constitutes a common suppressor gene program driven by the immunomodulatory cytokine IL-27 and specific transcription factors. Transcriptional signatures of CD8+ T cells are associated with the prognosis of a variety of autoimmune diseases, especially systemic diseases. In T1D, the fatigue state of CD8 cells was promoted by anti-CD3 therapy (teplizumab) and was more pronounced in islet-specific CD8+ T cells of those who progressed slowly, suggesting a benefit in T1D as well. When the prognosis is good, the expression levels of these inhibitory receptors are upregulated. At the same time, the expression of other genes related to T-cell receptor and cytokine signaling pathways, chemotaxis, adhesion, and migration also changes (87).

Based on the above background, a number of therapeutic approaches have been proposed to prevent T1D using islet β cell antigen-specific T cells (87). Abdelsamed et al. proposed that the proportion of beta cell-specific CD8+ T cells is inversely proportional to the progression of T1D, possibly due to the ability of these cells to maintain epigenetic programs associated with stem cell memory, enabling the maintenance of effect response (13, 40). However, this inverse relationship may only apply to exhausted CD8+ T cells, and CD8+ T cells may kill beta cells through other pathways, such as MHC1 (88) and other cytokines produced in the pancreas (89, 90). Studies have shown that T-cell populations characterized by both inhibitory receptors and depletion mediated by them are closely related to improved T1D markers (72). In T1D, anti-CD3 therapy promotes CD8 depletion, especially in islet-specific CD8+ T cells in slowly progressive patients (87). Interestingly, in T1D, TILs share common features with exhausted CD8+ T cells and are often dysfunctional, limiting antitumor immunity (79, 91). A more in-depth comparison with the “Exhausted” phenotype of cancer may help us understand the potential and limitations of T1D treatment. The exhausted T-cell phenotype in cancer has been extensively studied in immunotherapy, which includes the use of anti-PD-1 or anti-PD-L1 antibodies to unblock T cells. By comparing exhausted T-cell phenotypes in T1D and cancer, we can assess whether similar immunotherapy strategies can be applied to improve T1D therapy, such as enhancing exhausted T-cell reactivity through treatments targeting the PD-1/PD-L1 pathway. However, further research is needed to gain insight into the mechanism and function of exhausted T cells in T1D, as well as the differences and similarities with exhausted T cells in cancer. This will help develop more effective immunotherapy strategies to improve treatment response and disease management in patients with T1D. However, T-cell exhaustion is a double-edged sword for receptor characterization (87).

## 4 CD8+ T-cell receptors

Although T1D can be predicted today by measuring autoantibodies against β cell antigens in peripheral blood, there is an urgent need to develop T-cell markers to explain T-cell activity in the pancreas and to serve as a measure of disease activity (92). In recent years, immunosequencing and transcriptional profiling of the α/β chain complementary determinant of TCR have revealed the potential of TCR-altering biomarker discovery (93). In addition, a growing number of studies have shown that TCR signaling influences multiple aspects of CD8+ T-cell immunobiology, including thymus development, peripheral homeostasis, effector subset differentiation, function, and memory formation (94). Maladjusted TCR signaling events in T1D affect the efficacy of central and peripheral tolerance induction mechanisms, which also supports this idea (94).

**Figure 3:**
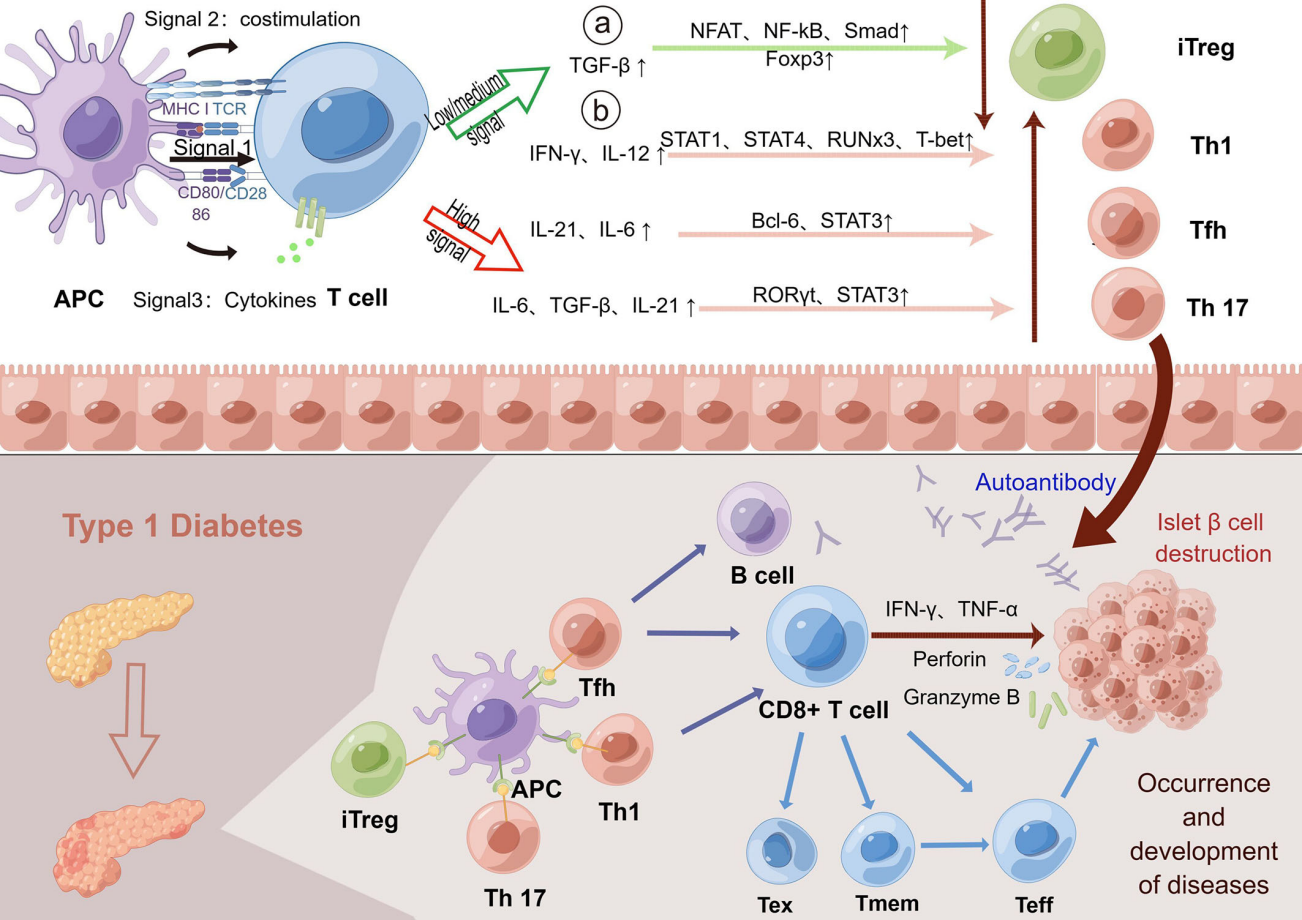
TCR indirectly promotes the development of T1D through several signaling pathways. The activation and differentiation of T cells depends on the strength of the interaction between TCR signaling, costimulatory signals and cytokines. Low TCR signaling can promote the differentiation of T cells into adaptive regulatory T-cell subsets by affecting the number of intermediate cytokines. Regulatory T cells help to regulate autoimmunity and prevent excessive autoimmunity. In contrast, high or persistently open TCR signaling promotes T-cell differentiation into immune-promoting Th1, Tfh, and Th17 subsets. In the normal human body, the body maintains autoimmune balance by regulating the opening of three pathways. However, in T1D patients, the continuous interaction of T cells with the MHC-antigen receptor compl_ex_ and/or enhanced signaling results in the continuous enhancement of TCR signaling to promote the generation of Th1, Tfh and Th17 subsets. Next, high TCR signaling can combine with costimulation and proinflammatory cytokines to promote the growth and differentiation of effector T cells and promote autoimmunity, which then spontaneously migrate to the islet to destroy islet β cells, leading to the development and progression of the disease. Low or moderate intensity TCR signaling can promote the transformation of T cells into exhausted T cells and hinder the function of effector T cells. However, elevated levels of the cytokine IL-21 promote the recovery of exhausted T cells and continue to cause islet β cell destruction. Memory T cells can also rapidly transform into effector T cells and produce proinflammatory factors in response to antigens to enhance the immune response, indirectly leading to the destruction of β cells. In conclusion, the increased TCR signaling in T1D could promote autoimmunity through multiple mechanisms, leading to islet β cell destruction and T1D. TCR, T-cell receptor; T1D, type 1 diabetes; Foxp3, forkhead box protein P3; iTreg, adaptive regulatory T cells; MHC, major histocompatibility complex; IA-2, islet antigen 2; GAD, glutamic acid decarboxylase.

Many studies have shown that TCR is closely related to the occurrence and development of T1D (40). The MHC region is a key factor in the genetic determination of both diabetes and other autoimmune diseases at the sites identified thus far. HLA mutations affect T-cell receptor (TCR)-mediated peptide binding and signal transduction, and how this affects antigen presentation is a crucial step in thymic selection and peripheral activation of immune responses. Additionally, the tissue specificity and progression of T-cell-mediated autoimmune responses depend in part on the TCR library expressed by pathogenic effector CD8+ cells (58). Other studies have shed light on the underlying mechanisms. It has been shown that alterations in TCR signaling (Signal 1), costimulatory signals (Signal 2) and cytokines (Signal 3) lead to impaired central and peripheral tolerance induction mechanisms, which ultimately lead to T-cell activation, amplification, and subpopulation differentiation induced by β cell autoantigens driven by epigenetic and transcriptional outcomes that promote proinflammatory responses (94). TCR signaling is responsible for the development and progression of T1D, which (88) helps explain the association of certain HLA genes with T1D susceptibility. Moreover, dysregulation of costimulatory signals and/or cytokines also drives alterations in the signaling outcomes of specific T cells that recognize β cells, which aid in the growth and differentiation of effector and memory T cells (effector T cells can differentiate into memory T cells) and inhibit the formation and differentiation of exhausted T cells and protective FoxP3-regulated T-cell responses. Therefore, we can conclude that changes in signaling pathways involving TCR may promote or inhibit the development of T1D, which is manifested by differences in the islet T-cell infiltration rate (94). Animal experiments have provided relevant evidence. Although the diversity of TCRβ species in conventional and nTregs in NOD mice was significantly inconsistent compared with that in C57BL/6 mice, the difference was more pronounced in TCRα (23). Takuro Okamura et al. used the latest technology of single-cell V (D)J sequencing in BD Rhapsody to identify the TCR sequences of the characteristic autoimmune T cells of T1D in Japan and found that the TCR diversity and gene expression differences of CD8+ and FOXP3+ cells were in patients with T1D and healthy subjects (95). The observed bias refers to the fact that certain amino acids are used more frequently than others in some specific T-cell receptor (TCR) sequences found in studies, a phenomenon known as TCR sequence bias. Codina-Busqueta et al. found that amino acid preferences in the NDN region revealed the presence of slanted TCR libraries in infiltrating T cells and that the monoclonal amplified TCR sequences contained amino acid combinations consistent with the observed bias (96). The bias observed in the study refers to the fact that in some specific TCR sequences found in pancreatic tissue from human T1D patients, certain amino acids in the NDN region are used more frequently than others, indicating a bias in the sequence of these T-cell receptors. The β chain of TCR also shows abnormal shortening and library sharing in T1D (97). It is worth mentioning that TCR-like antibodies against a proinsulin-containing fusion peptide improved T1D in NOD mice (98). Based on the above evidence, the collection of large TCR data sets in both patients and nonpatients with T1D and in combination with big data analytics will advance the development of TCR as a potentially powerful biomarker in the development of T1D (**Figure 3**) (92).

## 5 ScRNA-seq in T1D

### 5.1 The role of ScRNA-seq

There is still a lack of effective markers to monitor disease progression in T1D and to determine patient response to immunotherapy (99). The intensive use of flow cytometry methods has identified a variety of T cells that could serve as potential markers for T1D, such as FOXP3 IFN-γ Tregs (100), CD4+ T cells IL-2 response (101) and IL-21 CD4+ T cells (102). However, flow cytometry is very weak in discovering novel biomarkers, and it can only explore the possible role of existing markers in T1D. The high polymorphism and low precursor frequency of self-reactive T cells in PMBCs make it particularly important to accurately measure the RNA expression levels of complex mixtures of autoimmune T cells. Therefore, there is a need to tap more potential specific immune cell populations as markers for T1D development or prediction of prognosis, and single-cell RNA sequencing (scRNA-seq) can play an important role in this regard (103).

scRNA-seq can reveal information specific to individual cells in a cell population. sc-RNA-seq has later advantages: 1) sorting wells or using droplet-based techniques allows RNA heterogeneity or different spliceosomes of RNA in individual cells to be detected (104); 2) the V (D)J region of TCR and BCR can be detected, which allows studies to determine antigen specificity by paired TCRα and β strands (105); 3) in combination with protein expression, V (D)J sequencing can be used in conjunction with data such as standard gene expression to help uncover deeper pathogenesis or explore clinical value (106); and 4) compared with bulk RNA sequencing, scRNA-seq has less bias (107). Moreover, when using scRNA-seq, it is necessary to avoid a series of problems, such as small sample size, sample contamination, RNA interference from dying cells, and analysis errors, to obtain clear and reliable results.

More research strategies will be used in conjunction with scRNA-seq, e.g., spatial transcriptomics can compensate for the lack of spatial location information in single-cell sequencing (108). Cellular indexing of transcriptomes and epitopes by sequencing (CITE-seq) can provide cell surface protein expression. In combination with scRNA-seq, heterogeneous cells with similar gene expression and differential protein expression can be distinguished (109). The combination of machine learning and scRNA-seq can predict prognostic CD8+ T-cell characteristics (110). As scRNA-seq is explored more deeply, more potential markers will be explored, and more targeted drugs will be developed (**Table 2**).

**Table 2:** T1D-related single-cell sequencing study.

Model	Source	Marker	Results	Ref.
NOD mice	Pancreas and spleen	IGRP_206-214_	IGRP_206-214_ CD8+ T-cell have phenotypic heterogeneity and clonal restriction.	(15)
T1D patients	PBMCs	ZnT8_186-194_	ZnT8 + _186-194_ CD8+ T-cell clonotypes were found to cross-recognize a *Bacteroides stercoris* mimotope.	(25)
NOD mice	pLN	IGRP	IGRP CD8+ T cells with high expression of TCF1 in pLN induce T1D.	(76)
Human cells	B cell	ITGA1	develop a lineage model of *in vitro* β cell differentiation.	(111)
T1D patients	Pancreas	–	Establishing an electrophysiological and genetic link to islet dysfunction.	(112)
T1D patients	Pancreas	–	Sonic hedgehog signaling may regulateαcell proliferation.	(113)
NOD mice	Pancreas	DNMT1/ARX	DNMT1 and ARX maintain human α cell identity.	(114)
hESCs	hESCs	–	Activation of transcription factor NKX6.1 increases functional β cells.	(115)
NOD mice	islet	Tcf7	Tcf7 expression is not a critical determinant of T1D.	(116)
T1D rats	Splenocytes	–	MHC class II transcripts of monocytes and macrophages↑	(117)
T1DN mice	Podocyte	–	TRAP gene was differentially expressed in T1DN versus T2DN.	(118)
NOD mice	pancreas	CADM1	CADM1-mediated intercellular contacts promote the enrichment of CD8+ T cells in the pancreas.	(119)
T1D patients	PBMCs	CD45RA	Pediatric T1D is associated with CD45RA CD8+ T-cell population.	(120)
T1D patients	PBMCs	C1QB/NKG7	C1QB and NKG7 increase the number of macrophages and T cells, respectively, causing islet β cell injury.	(121)
T1D patients	PBMCs	T_H_1	Conversion of CD4+ cells to IFNG-T_H_1 memory phenotype in childhood.	(122)
T1D patients	PBMCs	PTPN6/TGFB/TYROBP	The regulatory genes PTPN6, TGFB, and TYROBP in DC form T1D↓	(123)
NOD mice	PBMCs	FoxP3/TGFβ1	FoxP3/TGFβ1+ CD4+ Tregs during autoimmune diabetes↓	(124)
T1D patients	PBMCs	–	Viruses and cytokines in pancreatic induce the release of IL-32 from activated T cells and NK cells in children.	(125)
T1D Patients	pancreas and PBMCs	–	snATAC-seq shows that risk variants for T1D were enriched in cCREs that were active in T cells.	(126)
T1D patients	T-cell	TCR	Specific antigens for CD8+ T cells were identified and TCRs cross-reacting with microbiome antigens were discovered.	(127)
T1D patients	PBMCs	TCR	islet Ag-reactive CD4+ Tregs are expanded during disease progression.	(128)
T1D patients	IAR T cells	TCR	The specificity of the IAR T-cell population is determined by the TCR.	(129)
Human	MPD	PDX1/ALK3/CAII	Progenitor-like cells in MPD can differentiate into functional β cells.	(130)
T1D patients	β cells and β-like cells	–	The genetic risk of T1D may be associated with β cell endoplasmic reticulum stress.	(131)
NOD mice	anti-insulin CD4+ T cells	TCR	Genetic correction of the I-Aβ57 mutation in T1D resulted in the loss of D/E residues in CDR3β of T cells.	(132)
T1D patients	PBMCs	BCR and TCR	Patients with type I diabetes have unique TCR and BCR-positive lymphocytes.	(133)
Human	Pancreas	–	Expression of an alternatively spliced INS product activated preproinsulin-specific CD8+ T cell.	(134
T1D patients	Pancreas	–	Expression of MHC class II pathway genes in exocrine ductal cells of T1D ↑	(135)

NOD, nonobese diabetic; pLN, pancreatic draining lymph node; IGRP, islet-specific glucose-6-phosphatase catalytic subunit-related protein; cCREs, candidate cis-regulatory elements; T1DN, type 1 diabetic nephropathy; TRAP, translating ribosome affinity purification; CADM1, Cell Adhesion Molecule 1; MPD, Myeloproliferative Disorder; TCR, T-Cell Receptor; PDX1, Pancreatic and Duodenal Homeobox 1; ALK3, Activin Receptor-Like Kinase 3; CAII, Carbonic Anhydrase II; C1QB, Complement C1q subcomponent subunit B; NKG7, Natural killer cell group 7 protein; BCR, B-cell receptor; PTPN6, Protein tyrosine phosphatase nonreceptor type 6; TGFB, Transforming growth factor beta; TYROBP, TYRO protein tyrosine kinase binding protein; DNMT1, DNA methyltransferase 1; ARX, Aristaless related homeobox; FoxP3, Forkhead box P3; TGFβ1, Transforming growth factor beta 1; ZnT8, Zinc transporter 8.

### 5.2 T1D-related ScRNA-seq study

ScRNA-seq can reveal the mechanisms of islet cell changes in T1D, for example, developing a linear model of β cell differentiation *in vitro* (111, 136), establishing the link between electrophysiology and genetics of islet dysfunction (112), and mining more specific loci that induce α cell or β cell proliferation and function (Sonic hedgehog signaling, DNMT1, ARX and transcription factor NKX6.1, etc.) (113–115). More importantly, it can tap more specific CD8+ T cells to construct models for diagnosis or determining prognosis and guide more precisely targeted therapies.

ScRNA-seq identifies specific subpopulations that influence T1D progression and can establish a link between cellular function and genetic modifications, ultimately guiding the use or modification of specific T cells for therapy. A subpopulation of IGRP stem cell-like CD8+ T cells in the pLN highly expresses TCF1, which may induce T1D (76). Other studies have shown that IGRP_206-214_ stem cell-like CD8+ T cells are phenotypically heterogeneous and clonally restricted (15) and that TCF1 is not a critical determinant of T1D (116). It has now been shown that H9T treatment maintains T-cell transcription factor 1 (TCF-1) expression and promotes mitochondrial adaptation, thus facilitating the maintenance of a stem cell-like state. Furthermore, TCR transgenic and chimeric antigen receptor-modified stem cell-like CD8+ T cells amplified with H9T cells showed potent *in vivo* antitumor activity in mouse models of melanoma and acute lymphoblastic leukemia (137). This offers hope for the future use of transgenic stem cell-like CD8+ T cells for the treatment of T1D.

Many more specific T-cell subsets were screened, providing additional insight into the mechanisms of CD8+ T cells in T1D. Following infection with Kilham rat virus (KRV), T1D onset was preceded by KRV invasion into multiple splenocytes, a reduction in CD8+ T-cell numbers, and scRNA-seq results showing increased expression of MHC class II transcripts on monocytes and macrophages in type I IFN and IFN receptor (IFNAR)-loss rats. IFNAR may be associated with T1D susceptibility (117). Podocyte-based scRNA-seq showed that the translation ribosome affinity purification (TRAP) gene was differentially expressed in T1D versus T2D (118). Cell adhesion molecule 1 (CADM1)-mediated intercellular contacts promoted enrichment of CD8+ T cells in the pancreas (119). Pediatric T1D was associated with naïve and activated/memory CD45RA CD8+ T-cell populations (120). C1QB and NKG7 increase the number of macrophages and CD8+ T cells, respectively, leading to islet β cell injury (121). In pediatric T1D, CD4+ cells were converted to the IFNG-T_H_ 1 memory phenotype (122). The regulatory genes PTPN6, TGFB and TYROBP inhibit DC in T1D (123). FoxP3/TGFβ1+ CD4+ T_reg_ cell number and function are decreased in T1D (124). It has also been noted that viruses and cytokines in the pancreas induce the release of IL-32 from activated T cells and NK cells in children (125). Notably, genome-wide association studies (GWAs) combined with single-cell epigenomics will play a role in understanding the complex origins of T1D. Risk variants in T1D were found to be enriched in candidate cis-regulatory elements (cCREs) active in T cells. Multiple potential risk variants in T1D overlap with cCREs in genes with exocrine-specific expression (126). Currently, many relevant sites on GWAs have been found to associate with different nodes in T-cell activation and signaling pathways (138).

### 5.3 ScRNA-seq of TCR

The single-cell transcriptome + TCR sequencing solution can obtain gene transcript levels at the single-cell level of resolution along with TCR light and heavy chain sequence information (139). Single-cell technology bridges the shortcomings of traditional TCR detection technology and provides the possibility of rapid screening of CD8+ T-cell targets in T1D immunotherapy. Combining TCR sequencing with T-cell phenotyping can yield more realistic insights into islet antigen-specific CD8+ T cells, which can separate CD8+ T cells that are actually involved in killing β cells, rather than being limited to having islet antigen binding sites.

High-dimensional tetramer-associated TCR-seq allowed the identification of specific antigens for CD8+ T cells and revealed antigenic cross-reactivity between TCRs and the microbiome in T1D (127). For example, the restriction zinc transporter protein 8 (ZnT8) 186-194 CD8+ T_eff_ cell clonotypic antigen can cross-recognize a *Bacteroides stercoris* mimotope (25, 140). The mechanism of TCR action in CD8+ T cells is gradually being uncovered by TCR sequencing. In an earlier study, islet antigen-reactive (IAR) CD4+ Tregs were found to be increased in T1D and could be diagnosed or predicted by peripheral blood scRNA-Seq assay (128). The specificity of IAR CD4+ T cells has been shown to be closely related to the self-response-restricted TCRα chain and its associated epitopes (129). The latest study used CRISPR/Cas9 to knock in PTPN22, a gene associated with altered TCR regulation and T-cell activation, in naive T cells, ultimately enhancing self-reactive T cells and shifting the differentiation of this subpopulation toward an inflammatory phenotype, demonstrating the possibility of altering the TCR and thus regulating inflammation through gene editing in T1D (141).

## 6 Potential targeting of CD8+ T cells for T1D treatment

### 6.1 CRISPR/Cas9 regulates CD8+ T cells

**Figure 4:**
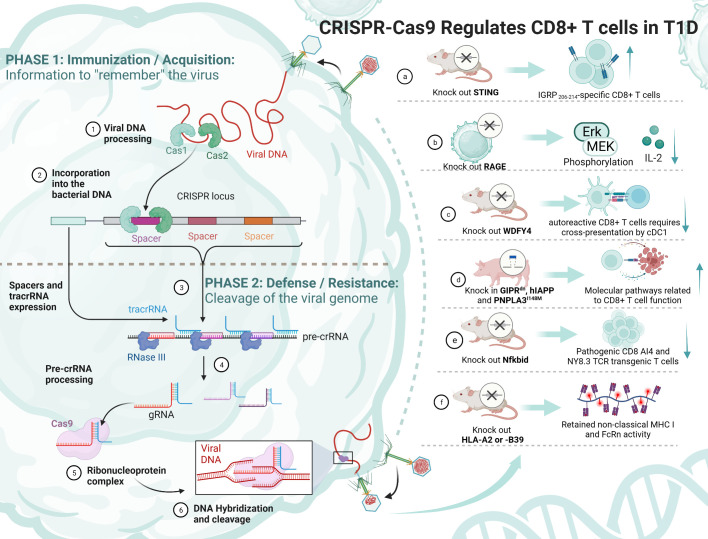
The principle of CRISPR/Cas9 and its mediation of T1D regulation by CD8+ T cells. TCF-1, transcription factor T cytokine 1; NOD, nonobese diabetic; RAGE, receptor for advanced glycation end products; WDFY4, WD repeat and FYVE domain-containing 4; cDC1s, type 1 conventional dendritic cells; STING, stimulator of IFN genes; IGRP, islet-specific glucose-6-phosphatase catalytic subunit-related protein; MHC, major histocompatibility complex.

CRISPR/Cas9 is a gene editing technology that was originally a bacterial defense mechanism against exogenous DNA (142). It performs target gene recognition through artificially designed guide RNA, directs Cas9 protease to break the DNA double strand, and causes genome modification during DNA damage repair. CRISPR/Cas9 enables gene knockout/knock-in, gene repression/activation, multiplex editing and functional gene screening. CRISPR/Cas9 technology has within just a decade been involved in solving many biomedical problems (143). The combination of two revolutionary technologies, immunotherapy and CRISPR/Cas9, has further broadened the application of immunotherapy in a variety of diseases. The role of CRISPR/Cas9 in T1D therapy is increasingly being explored, and an exhaustive summary is still lacking (**Figure 4**).

Some experiments have attempted to knock out a gene and thus achieve remission of T1D, but it is undeniable that its function and survival time are still immature and need to be explored in more depth (**Table 3**). Antigen activation of CD8+/CD4+ T_eff_ cells promotes receptor expression of the receptor for advanced glycation end products (RAGE), which may enhance T-cell reactivity and induce inflammation in T1D patients. One study using CRISPR/Cas9 knockdown of RAGE in Jurkat cells resulted in Erk, phosphorylation of MEK and reduced IL-2 (145). Knocking out WDFY4 using CRISPR/Cas9, thereby eliminating cross-presentation of type 1 conventional dendritic cells (cDC1s), was found to limit the initiation of self-reactive CD8+ T_eff_ cells in T1D and attenuate the recruitment of CD4+ T cells into islets to damage β cells. When the stimulator of IFN genes (STING) was knocked out in NOD mice, the number of IGRP _206-214_-specific stem cell-like CD8+ T cells was increased, and splenocytes from STING-deficient mice could be rapidly induced in T1D after relay transfer to irradiated NOD recipients (147). This study suggests that stem cell-like CD8+ T cells in T1D may be regulated by the STING pathway that senses DNA damage and, in the future, may be activated by activating the cGAS-STING pathway in stem cell-like CD8+ T cells to improve T1D. Earlier studies have shown that Nfkbid, an allelic variant of an NF-κB signaling regulator, induces thymic deficiency defects in self-reactive CD8+ T_eff_ cells in NOD mice with abrupt onset of T1D (149). The latest study enlarged thymic deficiency in pathogenic CD8 AI4 and NY8.3 TCR transgenic T cells by knocking in Nfkbid, but the study truncated the increased frequency and function of peripheral Treg, which inhibited the accelerated progression of T1D (151).

**Table 3:** CRISPR/Cas9 regulates CD8+ T cells in T1D.

CRISPR/Cas9 editing sites	Model	Express	Results	Ref.
TCF-1	rhesus macaque	↑	TCF-1 contributes to the maintenance of stem cell-like HIV-specific CD8+ Tmem cells.	(144)
RAGE	Jurkat cell	↓	Erk phosphorylation and IL-2 in CD8+ T-cell↓	(145)
WDFY4	NOD mice	↓	the priming of Autoimmune CD8+ T cells requires cross-presentation by cDC1↓	(146)
STING	NOD mice	↓	IGRP_206-214_-specific CD8+ T cells↑	(147)
GIPR^dn^, hIAPP and PNPLA3^I148M^	pig	↓	Molecular pathways related to CD8+ T-cell function↑	(148)
Nfkbid	NOD mice	↓	Spontaneous diabetic AI4 and NY8.3 CD8+ T-cell negative selection↓	(149)
HLA-A2 or -B39	NOD-cMHCI mice	↓	Retained nonclassical MHC I and FcRn activity	(150)

TCF-1, transcription factor T cytokine 1; NOD, nonobese diabetic; RAGE, receptor for advanced glycation end products; WDFY4, WD repeat and FYVE domain-containing 4; cDC1s, type 1 conventional dendritic cells; STING, stimulator of IFN genes; IGRP, islet-specific glucose-6-phosphatase catalytic subunit-related protein; MHC, major histocompatibility complex.

In a study using transgenic pigs as a model, knock-in of GIPR^dn^, hIAPP and PNPLA3^I148M^ revealed that CD8+ T cells in the liver and adipose tissue, costimulation, cytotoxicity and increased secretion of cytokines and chemokines, antigen presentation, and TCR signaling were all activated (148). This study suggests the possibility of knocking in pathogenic risk genes to construct a large animal model highly similar to T1D patients. Another study constructed NOD-cMHC I mice by knocking out the genes of human disease-associated HLA-A2 or -B39, which compensated for the previous lack of nonclassical MHC I molecule expression and FcRn activity in NOD.β2 m mice (150). There are also studies trying to find the use of CRISPR/Cas9 to construct stem cell-like CD8+ T cells. TCF-1 expression in CD8+ T cells correlates with memory marker expression and amplification capacity, and sustained antigenic stimulation decreases TCF-1 expression. In an experiment on HIV, CRISPR/Cas9 was used to knock in TCF-1 in CD8+ T cells, which possesses stem cell-like memory properties with secondary amplification capacity (144). It has been shown that a small number of stem cell-like CD8+ T cells with high TCF-1 expression greatly induces T1D. CRISPR/Cas9 technology might be able to generate stem cell-like CD8+ T cells that do not kill pancreatic islet cells, thus ameliorating T1D. These models could provide a broader direction for the development of future T1D therapies.

### 6.2 Chimeric antigen receptor T-cell

**Figure 5:**
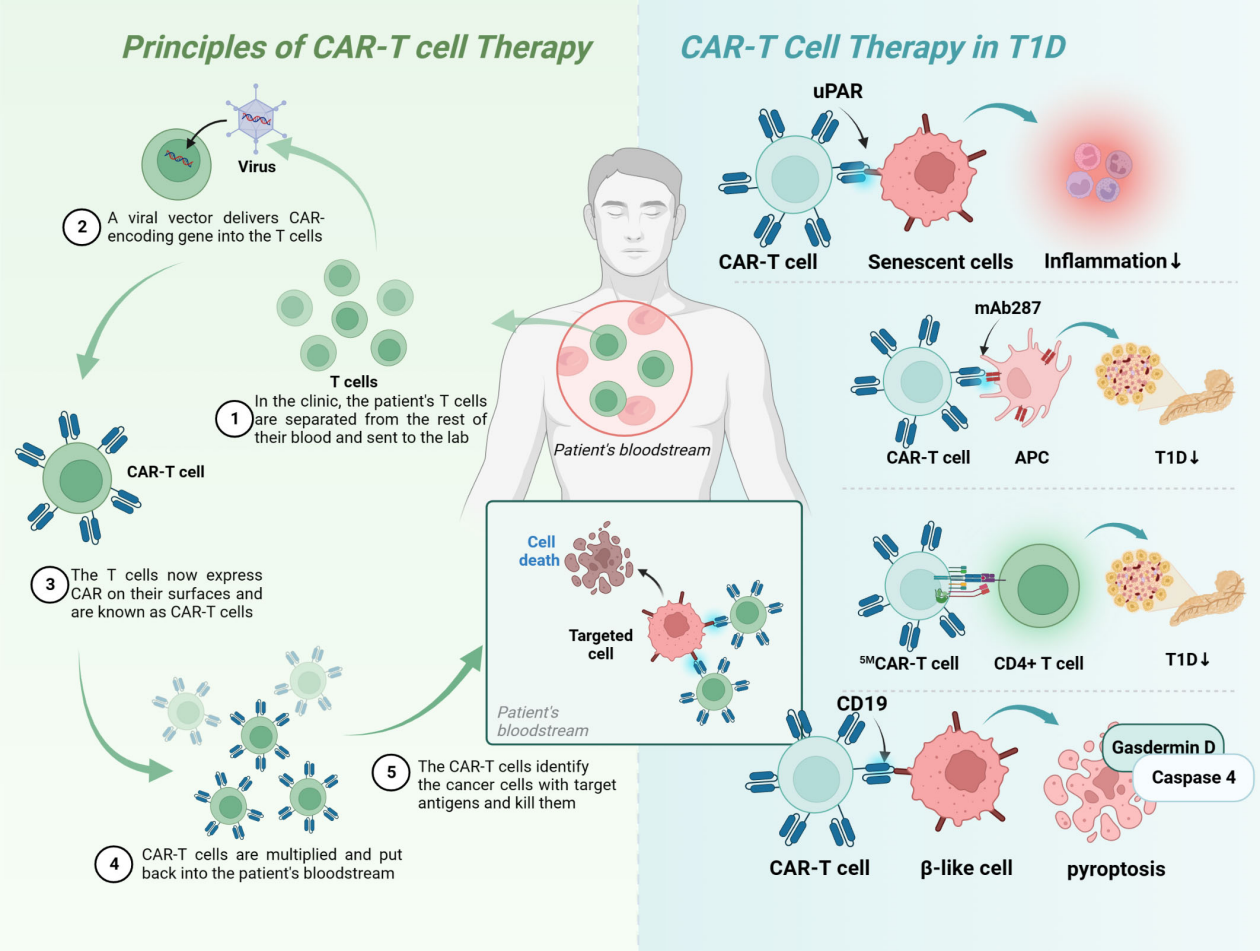
Principles of CAR-T-cell therapy and its application in T1D. Upar, urokinase-type plasminogen activator receptor; APC, antigen-presenting cells; MHC, major histocompatibility complex; T1D, type 1 diabetes; CAR, chimeric antigen receptor.

CAR-T therapy is a technology in which human T cells are extracted from the body, genetically engineered and cultured to a sufficient quantity, and infused back into the patient’s body to achieve a cure for the disease (**Figure 5**). Injecting Tregs into the body can alleviate the development of T1D, which is a promising novel therapeutic route (**Table 4**). However, only a small number of Tregs can be isolated, so an early study attempted to convert T_eff_ into T_reg_ using CAR-T technology, and the results showed that CAR-Tregs performed well in terms of function and duration of presence (155). More studies have been conducted to develop site-specific CAR-T cells for the treatment of T1D. CAR-T cells with urokinase-type plasminogen activator receptor (uPAR) specificity can target senescent cells to alleviate inflammatory responses and improve tissue homeostasis, which may improve diabetes, and future studies in NOD mouse models or human-derived transgenic models are needed to verify this (152). mAb287 is a monoclonal antibody that can target I-A binding between the insulin B chain 9-23 peptide and NOD MHC class II molecules. CAR-T cells formed by spiking CD8+ Teff cells with mAb287 can mediate the IFN-γ pathway to kill antigen-presenting cells (APCs) (153). However, a single infusion can only delay the development of T1D for 1-2 weeks, and more studies on optimizing the lifespan of CAR-T cells are needed in the future. A biomimetic study used CD3γϵ, δϵ, ζζ, CD4 and CD8+ to construct a biomimetic five-module chimeric antigen receptor (^5M^CAR), which drives CD8+ T-cell activation and binds MHC molecules. Experiments using NOD mice as a model showed that ^5M^CAR-T cells could alleviate T1D by targeting autoimmune CD4+ T cells (154). CAR-T technology can be used not only to treat T1D but also to explore potential mechanisms of CD8+ T-cell destruction. A study in which CD19 CAR-T cells were constructed explored the potential mechanisms of β cell damage that may be associated with factors such as scorch death and endoplasmic reticulum stress, where CD19 CAR-T cells release T-cell factors and upregulate immune response genes and the scorch death mediator Gasdermin D and its activator Caspase 4 in β-like cells, ultimately leading to the death of damaged β-like cells (157).

**Table 4:** CAR-T-cell therapy in T1D.

Model	Targeted Cell	Targeted proteins	Results	Ref.
Mice	senescent cells	uPAR	eliminating senescent cells from damaged tissues↑	(152)
NOD mice	APC	mAb287	Short-term prolonged onset of hyperglycemia	(153)
NOD mice	CD4+ T-cell	pMHCII	T1D↓	(154)
NOD mice	β cell	insulin	Insulin-specific CAR Tregs is functionally stable *in vivo* and has long-term inhibitory properties.	(155)
Mice	–	LFA-1	Low serum magnesium levels reduce CAR-T-cell function and activity.	(156)
β-like cell	β cell	CD19	Immune response gene and scorch death mediator Gasdermin D and its activator Caspase 4 in β cell ↑	(157)

Upar, urokinase-type plasminogen activator receptor; APC, antigen-presenting cells; MHC, major histocompatibility complex; T1D, type 1 diabetes; CAR, chimeric antigen receptor.

Treatment with CAR-T-cell technology alone may not yield the desired benefits for patients, with several drawbacks: 1) the high time and economic cost of producing individual-specific CAR-T cells to rescue rapid disease progression; 2) the inability to obtain sufficient raw material when patients have fewer high-quality lymphocytes; and 3) the clinical architecture produced by autologous CAR-T cells can become uncontrollable and unpredictable because the heterogeneity of autologous CAR-T cells can become uncontrollable and unpredictable (158). The CRISPR/Cas9 system mentioned above could improve or consolidate the effect of CAR-T-cell and TCR-T-cell technologies, for example: 1) the construction of universal CAR-T cells with knockdown of TCR and HLA molecules, which would make future immunotherapy with adoptive T-cell transfer (ACT) timely and standardized; 2) the use of CRISPR/Cas9 knockout/in immune checkpoint genes; and 3) or knockout of T-cell factor genes, which may improve treatment outcomes (159).

## 7 Future perspectives

**Figure 6:**
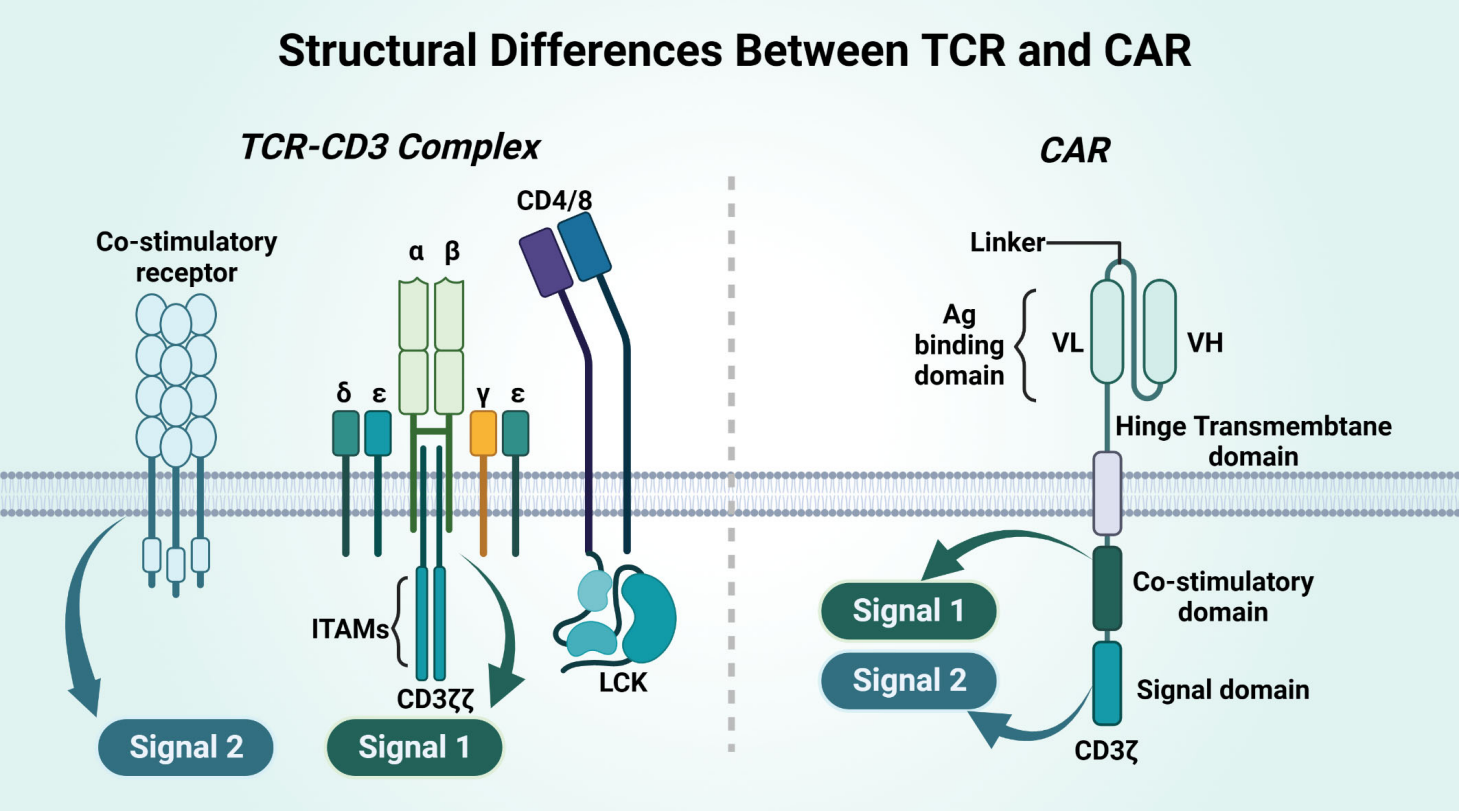
Structural differences between TCR and CAR.

TCR-T cells have a highly specific TCR αβ chain sequence, which can recognize antigens inside cells compared to CAR-T cells (**Figure 6**). The process is roughly as follows: 1) screening and identification of TCR sequences that can specifically bind to target antigens; 2) genetic engineering to transfer them into T cells and culture them to sufficient numbers; and 3) transfusion of TCR-T cells back into patients to specifically recognize and kill cells for therapeutic purposes (160, 161).

CAR and TCR have different structures, and their affinities and their effects differ (**Table 5**). CAR is an artificial single chain with five structural domains. The variable heavy chain (VH) and variable light chain (VL) structural domains combine to form an antigen-binding structural domain (scFv) that binds cytokines or ligands significantly beyond the TCR (171). The costimulatory domain and the CD3ζ signaling structural domain activate T cells, and the hinge domain and TM domain are involved in the formation of CAR-T-cell immune synapses (167, 168). The costimulatory domain transmits both signal 1 and signal 2, on the basis of which T cells will be completely activated. TCR binding to the homologous peptide-MHC complex (pMHC) does not complete signaling directly but requires complex formation with multiple CD3 signaling subunits for T cells. CD3γϵ and CD3δϵ and CD3ζζ together form the TCR-CD3 complex, and the extracellular immunoglobulin (Ig) superfamily structural domain, based on the activation motif of the immune receptor tyrosine (ITAM), in which the extracellular structural domain (ECD) is contained, will complete Signal 1 alone. Signal 2 of TCR-T cells requires costimulatory receptor-ligand binding of 4-1BB and 4-1BBL for this to occur, and Signal 2 synergizes with Signal 1 to fully activate T cells (172).

**Table 5:** Differences between TCR-T and CAR-T cells.

Elements	CAR-T	TCR-T	Ref.
Structure	Single chain with 5 structural domains	TCR-CD3 complex	(162)
Antigen expression	Cell surface	Inside and outside the cell	(160)
MHC restriction	Not restricted	Restricted	(163)
ITAMs	10	3	(164)
Immune rejection	Low	High	(165)
Permeability	High	Low	(166)
Coreceptor involvement	CD45	CD4, CD8 and CD45	(167, 168)
FDA approval	Kymriah, Yescarta, Tecartus, Breyanzi, Abecma, Carvykti	Tebentafusp	(169, 170)

There are six CAR-T therapies that have been approved by the FDA and have shown some success in their therapeutic use for the treatment of hematologic malignancies (170). There is currently only one FDA-licensed TCR-T therapy drug: tebentafusp (169). At the same time, factors such as lack of specific target antigens and immunosuppression of the tumor microenvironment have been exposed in clinical applications. In contrast, the mechanistic exploration and clinical conclusions of TCR-T therapies are not sufficient, but there is some evidence that engineered TCR-T cells have better expansion rates within the high antigen environment, lower levels of coinhibitory immune checkpoint molecule expression, higher differentiation trends and no significant difference in tumor cell clearance (173).

ScRNA-seq and CRISPR/Cas9 in combination with TCR-T therapy is a promising strategy. ScRNA-seq technology can identify specific T-cell clones at the single-cell level to help obtain high-affinity TCR sequences or analyze the activity of TCR-T cells *in vivo* to guide therapy (160). The presence of endogenous TCRs on the surface of CD8+ T cells to be modified is a major factor affecting the therapeutic efficacy of TCR-T cells. Endogenous TCRs lead to a reduced binding rate of transgenic TCRs to CD3, and the two may mismatch and form mixed TCR dimers. To address the problems of a low transgenic TCR-CD3 binding rate and mispairing, CRISPR/Cas9 can transduce a stable Vα/Vβ single-stranded TCR into T cells. Alternatively, CRISPR/Cas9 can replace endogenous TCR α and β genes with genes having certain specific TCR sequences to better ameliorate the disease. In addition, it has been shown that transgenic TCR expression and function in TCR-T cells without knockdown of endogenous TCR genes is lower than that in TCR-T cells with knockdown of endogenous TCR (174, 175). CRISPR/Cas9-edited TCR-T cells were tested in clinical trials in patients with refractory cancers to assess safety and feasibility. Of the three patients treated, two had stable disease, and the other had progressive disease without serious adverse effects (176).

In addition, most of the CAR/TCR-T experiments have been realized only in tumor models, and it remains to be explored whether CAR/TCR-T cells have the real ability to be used as a T1D treatment targeting autoimmune remission based on more NOD mice or even clinical experiments. The targeting toxicity, neurotoxicity and cytokine storm generated (CSG) by CAR/TCR-T cells are also issues to be overcome in the future.

## 8 Conclusion

This review suggests that self-reactive CD8+ T cells have great potential in the treatment of T1D and that targeted precision therapy may be possible by combining multiple novel technologies. The main existing therapies for T-cell-mediated T1D include systemic immunosuppression, antibodies that deplete immune cells, and anti-cytokine therapies. Although effective in reducing autoimmune T cells, they may also impair other immune responses, leading to increased susceptibility to other diseases and complications. In contrast, identifying specific CD8+ T cells by scRNA-seq technology with TCR sequencing and modifying them by technologies such as CRISPR/Cas9, CAR-T, and TCR-T, making it possible to cure T1D by losing the ability to recognize β cells to β cell-specific stem-like CD8+ T cells and its differentiated progeny, is a potential pathway. In addition, scRNA-seq and CRISPR/Cas9 may also play an important role in the evaluation of therapeutic efficacy and construction of experimental models in the future. It is worth noting that many studies have only measured the number of CD8+ T cells, but have not focused on their specific exhaustion, stem cell-like and other state changes, which is something that future studies need to focus on to analyze the mechanism of CD8+ T cells in T1D from a more dynamic perspective.

In the future, many more factors need to be considered: 1) the effect of pathogenic single nucleotide polymorphisms (SNPs) on T-cell function; 2) activation of T cells by unnatural peptides, posttranslationally modified peptides, and hybrid peptides; and 3) the interaction between B cells and T cells and DC cells and T cells. Mendelian randomization, molecular simulation, organoids and other techniques will bring newer perspectives to the field.

## Author contributions

KY: Writing – original draft, Writing – review & editing. YZ: Writing – original draft. JD: Writing – original draft. ZL: Visualization, Writing – original draft. HZ: Visualization, Writing – original draft. FZ: Funding acquisition, Writing – review & editing.
